# Suicide prevention curriculum development for health and social care students: Protocol for a scoping review

**DOI:** 10.1371/journal.pone.0285231

**Published:** 2023-12-07

**Authors:** Kerrie Gallagher, Clíodhna O’ Brien, Michelle O’ Driscoll, Doireann Ní Dhálaigh, Paul Corcoran, Eve Griffin

**Affiliations:** 1 National Suicide Research Foundation, Cork, Ireland; 2 School of Public Health, University College Cork, Cork, Ireland; Lahore Medical and Dental College, PAKISTAN

## Abstract

Suicide has become a serious public health concern and international research has shown that the majority of individuals who die by suicide had receive healthcare in the year prior to their death. This presents an opportunity for suicide prevention by strategically training healthcare students in suicide prevention knowledge and skills. The objective of this scoping review is to identify literature that describes the design, development, implementation and/or evaluation of suicide prevention training for healthcare and/or social care students in higher education settings. Studies will only be considered eligible for inclusion if they describe the design, development, implementation and/or evaluation of suicide prevention curricula being delivered to healthcare or social care degree students in higher education. Quantitative, qualitative, and mixed method studies published between 2011 and 2023 (inclusive) and in the English language will be considered eligible. This scoping review will be conducted according to the PRISMA guidelines for scoping reviews (PRISMA-ScR). The developed search strategy will be implemented across six databases: PubMed, ERIC (Education Resources Information Center), PsycINFO, Embase, CINAHL and Web of Science. Several grey literature sources will also be consulted. Further potential results will be located by hand-searching the reference lists of included articles. The search strategy will include variations of the terms: ‘*student*’, ‘*suicide prevention*’ and ‘*education*’. The search will be limited to titles, abstracts, and keywords in databases that allow it. Two reviewers will complete the screening using the predefined inclusion criteria. A third reviewer will resolve any conflicts during the screening and eligibility appraisal processes. Results will be presented in the form of tabulated results and an accompanying narrative summary, describing key findings and context related to learning outcomes, methodologies employed and implementation of the identified programmes.

## Introduction

It is widely recognised that suicide is a serious public health concern, with more than one in every hundred deaths attributed to suicide in 2019 [1]. In Ireland, suicide is reported to be the cause of approximately 500 deaths each year. However, this number is likely to be underestimated as a result of misclassification and overly high burdens of proof required to register a suicide death [2]. Importantly, there is now an increased understanding of suicidal behaviour based upon the "iceberg” model of self-harm [3, 4]. In this model, the incidence of suicide is represented at the tip of the iceberg, followed by higher incidences hospital-treated self-harm, and at its very base, the more common, yet hidden cases of self-harm that have not been observed/treated [3, 4].

This provides a unique opportunity to incorporate suicide prevention strategies in healthcare settings. These strategies can focus on providing mental health support, screening for suicidal risk, early intervention, and creating an environment that encourages help-seeking behaviour. However, evidence shows that many existing clinical training programmes do not adequately prepare healthcare professionals to engage in suicide prevention [5, 6].

A population-based cohort study conducted in Sweden between 2004 and 2015 found that 86% of individuals who died by suicide received healthcare in the year preceding their death [7]. It was also estimated that in the month before their death, 53% of those who died by suicide had received some form of healthcare consultation. This is compared with 20% in the general population [7]. Health professionals, such as nurses, pharmacists, primary care providers and social workers, are well-positioned to detect signs and symptoms of suicidal ideation and behaviours and provide those at risk with the appropriate referrals to support [8–11]. Training of healthcare staff has been highlighted as an important element of suicide intervention and prevention [12]. However, healthcare staff often do not receive appropriate training or lack knowledge and awareness of suicide and self-harm that allows for an appropriate response to suicide risk, primarily due to a lack of resources for healthcare professionals to gain access to appropriate information and training [13–15]. This is reflected in the fact that both professional practitioners and students rate their own interpersonal suicide prevention skills as poor [16]. Healthcare professionals also report a lack of confidence in their own competence in risk screening and intervention and this may serve as a barrier to effective suicide prevention in the healthcare setting [17, 18].

A suicide prevention programme targeted at healthcare and social care students provides an ideal opportunity to ensure a universal approach to suicide prevention in the healthcare system, as all graduates will begin their careers with similar knowledge, attitudes, and skills. In addition, a growing body of international evidence indicates that healthcare students may be at higher risk for poor mental health outcomes, suicidal ideation and behaviour [19–21]. For example, one cross-sectional study examining mental wellbeing and its associated factors among undergraduate pharmacy students from 14 countries (*n* = 2,665) found that more than one third of participants had poor mental wellbeing [21]. Thus, incorporating suicide prevention training in the curriculum of health and social care students may have a positive impact on help-seeking behaviours among this cohort through improved mental health literacy [22, 23].

To the best of the authors’ knowledge, the extent of the evidence regarding a suicide curriculum for health and social care students has not been reported on to date. The aim of this article is to describe the protocol for a scoping review to identify literature that describes the design, development, implementation and/or evaluation of suicide prevention training for healthcare and/or social care students in higher education settings. This question was prompted by the recognised need for a national standardised undergraduate suicide prevention curriculum in the Republic of Ireland as stated in Action 5.4.4 of *Connecting for Life*: *Ireland’s National Strategy to Reduce Suicide* [24]. It is intended that this scoping review will help inform the development of a suicide prevention curriculum for undergraduate health and social care students in the Irish higher education setting.

## Methods

The reporting items in this protocol comply with the Preferred Reporting Items for Systematic Reviews and Meta-Analyses extension for Scoping Reviews (PRISMA-ScR) guidelines which are presented [25] (see S1 File). We chose to adopt a scoping review approach for this research as the evidence on suicide prevention curricula is still emerging, particularly for health and social care students. A scoping review is an appropriate method for this research given that the main aim of this review is to identify studies that encompasses any aspects of the design, development, implementation and/or evaluation of suicide prevention training for healthcare and/or social care students in higher education settings. To identify potential similarities and differences between suicide prevention programmes, we intend to review the available evidence regarding the content and delivery of these programmes and, where applicable, their impact on student learning. We used the framework of Arksey and O’Malley to design the scoping review protocol [26] and utilised five primary stages to inform the study design: (i) identifying the research question; (ii) identifying relevant studies; (iii) study selection; (iv) charting data; (v) collating, summarizing, and reporting the results.

### (i) Identifying the research question

The scoping review will seek to address the following question:

‘What are current practices in the design, development, implementation and/or evaluation of suicide prevention training for healthcare and/or social care students in higher education settings?’

### Inclusion and exclusion criteria

Studies that examine suicide prevention training being delivered to undergraduate/postgraduate health and/or social care degree students in a higher education setting (i.e., colleges and universities) will be considered eligible for inclusion in the review process. All study designs will be included that examine any aspect of the design, development, implementation and/or evaluation of suicide prevention training for healthcare and/or social care students in higher education settings. Specifically, we will include any study which describes suicide prevention training delivered to higher education students who are studying to work in any of the following settings: disability, older persons & working age adult care settings, nursing homes, acute and non-acute hospitals, community hospitals, mental health, social inclusion, palliative care, chronic illness, primary care (GP, dental, pharmacies, physiotherapy clinics), health and well-being, hospice, rehabilitation, home care, paramedics, and community services (e.g. youth, substance abuse, suicide prevention, community development) [27]. Given the exploratory nature of the research, no control group is required in the studies, and we will include all studies that address any aspect of the design, development, implementation and/or evaluation of suicide prevention training for healthcare and/or social care students in higher education settings. There will be no limits set on region of publication, however, due to limited resources, studies published in the English language since 1^st^ January 2011 will be included for examination. Studies will not be included if they report on suicide prevention training implementation outside of the higher education curriculum for health and/or social care students (i.e., to health and/or social care professionals), are in a language other than English, and are published before 1^st^ January 2011. Given the exploratory nature of the research, we will not exclude any study design.

### Types of sources

Quantitative, qualitative, and mixed method studies, as well as any studies that describe the development of a suicide prevention module in higher education settings will be considered for inclusion. Any relevant grey literature will also be included.

### (ii) Identifying relevant studies

The search strategy will aim to locate peer-reviewed articles and any relevant grey literature, if available. An initial limited search of PubMed and Web of Science Core Collection was undertaken to identify the potential scope of published articles on the topic. The text words contained in the titles and abstracts of relevant studies, and the index terms used to describe the articles will be used to develop a full search strategy for the following electronic databases: PubMed, PsycINFO, ERIC, Web of Science Core Collection, CINHAL, EMBASE. It is anticipated that the search strategy for the review, including all identified keywords will be tailored to each database. The search will be limited to titles, abstracts, and keywords in databases that allow it. A manual search of the reference lists of the included studies will be conducted in order to identify any potentially overlooked materials, as well as a forward search for articles citing the articles identified by our search. Should conference abstracts and study protocols satisfy the inclusion criteria, we will endeavour to retrieve a peer-reviewed publication on the research which will be included. The search strategy will include variations of the terms ‘*students’*, ‘*suicide prevention’* and ‘*education*’. An example of the search strategy to be used specific to PubMed is provided in the S2 File. Further to this, relevant grey literature sources will be searched to retrieve items that would not otherwise be available through the above databases; several grey literature sources will be included in this project, including Google Scholar, the Suicide Prevention Resource Center (SPRC), BASE and ResearchGate.

### (iii) Study selection

Following the search, all identified citations will be collated and uploaded into the reference manager Zotero, Version 6.0.20. After this, all citations will be imported into Rayyan, and duplicates removed. Rayyan is a free web-tool designed to assist researchers conducting systematic reviews, scoping reviews, and other projects involving knowledge synthesis [28]. Screening will follow two stages: (i) title and abstract screening; (ii) full-text screening. Regarding the grey literature, which often lacks abstracts, texts will be screened through executive summaries, table of contents, or other comparable methods. If these methods of screening are not available, grey literature will be advanced to full-text screening. Two reviewers will independently conduct both screening stages and assess each stage against the inclusion criteria. In the event of any discrepancies, a third reviewer will be consulted. This search and study inclusion process will be described in full in the final scoping review along with a Preferred Reporting Items for Systematic Reviews and Meta-analyses extension for scoping review (PRISMA-ScR flow chart) [25]. Using the PRISMA diagram, the review process will be illustrated and stages where studies were eliminated will be recorded.

### (iv) Charting the data

A purpose-designed data extraction spreadsheet will be used to extract the following information from all included studies: Data items will include details of the publication e.g., author, country, year of publication, study setting (e.g., university); the study population (degree course, stage of the degree, number of students); details of the programme (e.g., session length, number of sessions); learning outcomes; accreditation body, was the training deemed to be ‘essential’ and was attendance ‘mandatory’; level of content delivered (i.e., foundational, basic, advanced, expert, other, unspecified); how the programme was implemented; methodologies used; staff training needs and assessment methods employed). A draft extraction form is provided in the S3 File. The draft data extraction tool will be modified and revised as necessary during the process of extracting data from the various evidence sources that have been included in the review. Any modifications made during this stage of the process will be detailed in the scoping review. If disagreements arise between the reviewers, they will be resolved by involving the third reviewer.

### (v) Collating, summarising, and reporting the results

The objective of this scoping review is to map existing evidence regarding the development and implementation of suicide prevention programmes in higher education. Data abstraction will be conducted by two reviewers independently from full-text records included in the review. As a means of ensuring accuracy and consistency, each reviewer’s abstracted data will be compared, and discrepancies will be discussed with the third reviewer. A request for missing or additional information will be made to the authors of selected records, if necessary. To facilitate the mapping of common patterns in the data, thematic analysis methods will be employed. This process will provide a summary of key findings regarding suicide prevention for health and social care students, including programme design and/or implementation. Where applicable changes in student knowledge, attitudes and confidence will also be reported. Any relevant abstracted information will be charted in the form of tabular results and an accompanying narrative summary, which will include key findings and contextual information regarding the topics, learning outcomes, and methodologies employed.

## Limitations

The authors recognise that this study will have limitations. As we are constrained by resources, only studies published in the English language will be included. We have also noted a limitation with respect to the search of grey literature; even if grey literature is reviewed systematically, some sources may be overlooked. We will attempt to overcome this by searching as many sources as possible in the time we have allocated to complete the review.

## Discussion

The interaction between patients and their healthcare professional provides an opportunity to screen and assess for patient suicide risk given the evidence that the majority of people who die by suicide have been in contact with a health professional in the year preceding their death [29]. The provision of suicide prevention training for students of the healthcare and social care professions presents an ideal opportunity to equip healthcare and social care professionals with relevant suicide prevention skills, knowledge, and confidence. The purpose of the proposed review is to give an overview of the design, development, implementation and/or evaluation of suicide prevention training for healthcare and/or social care students in higher education settings. The information gathered will be presented in a published scoping review.

The findings will be used by the authors in National Suicide Research Foundation with the Health Service Executive National Office for Suicide Prevention to inform the development of a national suicide prevention curriculum for all health and social care undergraduate students in Ireland. As a basis for designing the curriculum, the authors intend to develop a core-competency framework for suicide prevention. Through this framework, it is the authors desire to impart the necessary skills, knowledge and attitudes needed to develop an appropriate response to those in need. Moreover, it is hoped that a competency-based framework will help students understand the complexities of suicide prevention and reduce stigma surrounding this sensitive topic. Specifically, the results of this study are expected to support the development of a framework that includes intended learning outcomes, teaching methods, and supporting learning activities and assessments. For instance, results gained from this study may provide insight into the most appropriate strategies to teach the subject matter. These strategies may include using lectures, tutorials, and simulations/role plays, as well as the use of technology to help learners develop these fundamental skills.

## Supporting information

S1 File PRISMA-ScR template. (DOCX)

S2 File PubMed search strategy. (DOCX)

S3 File Data extraction table. (DOCX)
