# Supplementary material for: Suicide prevention curriculum development for health and social care students: Protocol for a scoping review
Source: PLoS One. 2023 Dec 7;18(12):e0285231. doi: 10.1371/journal.pone.0285231 (PMC10703193; doi:10.1371/journal.pone.0285231)
Supplement: S2 File — (DOCX) [file pone.0285231.s002.docx]

**Supplementary Files**

S2 File:

**PubMed Search Strategy and Results**

| Search number | Query | Filters | Results |
| --- | --- | --- | --- |
| 27 | #3 AND #17 AND #24 | English, from 2011 - 2023 | 424 |
| 26 | #3 AND #17 AND #24 | from 2011 - 2023 | 434 |
| 25 | #3 AND #17 AND #24 |  | 628 |
| 24 | #18 OR #19 OR #20 OR #21 OR #22 OR #23 |  | 412,912 |
| 23 | Postgraduate*[Title/Abstract] |  | 24,429 |
| 22 | Undergraduate*[Title/Abstract] |  | 54,502 |
| 21 | Student*[Title/Abstract] |  | 362,390 |
| 20 | "Social care student*"[Title/Abstract] |  | 58 |
| 19 | "Healthcare student*"[Title/Abstract] |  | 1,056 |
| 18 | "Students, Health Occupations"[Mesh] |  | 87,491 |
| 17 | #4 OR #5 OR #6 OR #7 OR #8 OR #9 OR #10 OR #11 OR #12 OR #13 OR #14 OR #15 OR #16 |  | 3,695,883 |
| 16 | Course [Title/Abstract] |  | 642,426 |
| 15 | Syllabus [Title/Abstract] |  | 1,068 |
| 14 | Program*[Title/Abstract] |  | 1,094,949 |
| 13 | Curricul*[Title/Abstract] |  | 73,634 |
| 12 | Learning [Title/Abstract] |  | 481,698 |
| 11 | "Learning"[Mesh] |  | 437,846 |
| 10 | Teaching [Title/Abstract] |  | 172,491 |
| 9 | "Teaching"[Mesh] |  | 93,136 |
| 8 | Training [Title/Abstract] |  | 553,525 |
| 7 | "Clinical Competen*"[Title/Abstract] |  | 4,479 |
| 6 | "Clinical Competence"[Mesh] |  | 105,319 |
| 5 | Educat*[Title/Abstract] |  | 787,804 |
| 4 | "Education"[Mesh] |  | 900,703 |
| 3 | #1 OR #2 |  | 15,116 |
| 2 | "Suicide Prevention*"[Title/Abstract] |  | 7,011 |
| 1 | "Suicide Prevention"[Mesh] |  | 11,343 |
