# Supplementary material for: Suicide prevention curriculum development for health and social care students: Protocol for a scoping review
Source: PLoS One. 2023 Dec 7;18(12):e0285231. doi: 10.1371/journal.pone.0285231 (PMC10703193; doi:10.1371/journal.pone.0285231)
Supplement: S3 File — (DOCX) [file pone.0285231.s003.docx]

**Supplementary Files**

S3 File:

**Data Extraction Table**

| **All Studies** | | | | | | | | | | | | | | | | | **Evaluation Studies Only** | | | | | |
| --- | --- | --- | --- | --- | --- | --- | --- | --- | --- | --- | --- | --- | --- | --- | --- | --- | --- | --- | --- | --- | --- | --- |
| Authors | Country | Setting | Degree course(s) | Stage of the degree | Student population | Number of students | Number of sessions | Session length | % of module dedicated to suicide prevention | Accreditation body | Is the training deemed essential or required? | Attendance mandatory | Did the staff require additional training? | How was the programme implemented? | What are the learning outcomes? | What methodologies were used? | Primary aim of the study | Main findings | Intervention group details | Type of control | Length of follow-up | Mean and standard deviation of all study groups for the relevant outcomes at all assessment times |
|  |  |  |  |  |  |  |  |  |  |  |  |  |  |  |  |  |  |  |  |  |  |  |
|  |  |  |  |  |  |  |  |  |  |  |  |  |  |  |  |  |  |  |  |  |  |  |
|  |  |  |  |  |  |  |  |  |  |  |  |  |  |  |  |  |  |  |  |  |  |  |
